# First human case report of sepsis due to infection with *Streptococcus suis* serotype 31 in Thailand

**DOI:** 10.1186/s12879-015-1136-0

**Published:** 2015-09-30

**Authors:** Rujirat Hatrongjit, Anusak Kerdsin, Marcelo Gottschalk, Dan Takeuchi, Shigeyuki Hamada, Kazunori Oishi, Yukihiro Akeda

**Affiliations:** Faculty of Science and Engineering, Kasetsart University Chalermphrakiat Sakon Nakhon Province Campus, Muang, Sakon Nakhon Province 47000 Thailand; National Institute of Health, Department of Medical Sciences, Ministry of Public Health, Tiwanon Road, Muang, Nonthaburi Province 11000 Thailand; Faculty of Veterinary Medicine, University of Montreal, Québec, Canada; Laboratory of Clinical Research on Infectious Diseases, International Research Center for Infectious Diseases, Research Institute for Microbial Diseases, Osaka University, 3-1 Yamadaoka, Suita, Osaka 565-0871 Japan; Thailand-Japan Research Collaboration Center on Emerging and Re-emerging Infections, Nonthaburi Province, Thailand; Infectious Disease Surveillance Center, National Institute of Infectious Diseases, Shinjyuku, Tokyo Japan; Present address: Faculty of Public Health, Kasetsart University Chalermphrakiat Sakon Nakhon Province Campus, Sakon Nakhon, Thailand

**Keywords:** *Streptococcus suis*, Serotype, Sepsis, Capsule locus

## Abstract

**Background:**

*Streptococcus suis* is a zoonotic pathogen that causes invasive infections in humans and pigs. It has been reported that *S. suis* infection in humans is mostly caused by serotype 2. However, human cases caused by other serotypes have rarely been reported. This is the first report of a human case of infection with *S. suis* serotype 31 in Thailand.

**Case presentation:**

A 55-year-old male alcohol misuser with liver cirrhosis was admitted with sepsis to a hospital in the Central Region of Thailand. He had consumed a homemade, raw pork product prior to the onset of illness. He was alive after treatment with ceftriaxone and no complication occurred. An isolate from blood culture at the hospital was suspected as viridans group *Streptococcus*. It was confirmed at a reference laboratory as *S. suis* serotype 31 by biochemical tests, 16S rDNA sequencing, and multiplex polymerase chain reaction for serotyping, but it was untypable by the co-agglutination test with antisera against recognized *S. suis* serotypes, suggesting loss of capsular material. The absence of a capsule was confirmed by transmission electron microscopy. The isolate was confirmed to be sequence type 221, with 13 putative virulence genes that are usually found in serotype 2 strains.

**Conclusion:**

We should be aware of the emergence of *S. suis* infections caused by uncommon serotypes in patients with predisposing conditions. Laboratory capacity to identify *S. suis* in the hospital is needed in developing countries, which can contribute to enhanced surveillance, epidemiological control, and prevention strategies in the prevalent area.

**Electronic supplementary material:**

The online version of this article (doi:10.1186/s12879-015-1136-0) contains supplementary material, which is available to authorized users.

## Background

*Streptococcus suis* is a zoonotic pathogen that causes invasive infections in humans who have been in close contact with infected pigs or contaminated pork-derived products, and this disease has lately received increasing attention worldwide [1]. Of the 29 serotypes that have been described, most of the clinical isolates from human cases are serotype 2 strains [1]. However, human cases caused by serotypes 1, 4, 5, 14, 16, 21 and 24, as well as an untypable strain, have been reported; in particular, serotypes 5, 14 and 24 and an uncapsulated strain have been found in Thailand [1–3]. Serotypes other than serotype 2 cause different clinical manifestations such as septic arthritis and peritonitis caused by serotype 5, meningitis by serotype 4 and 21, sepsis by serotype 24, and peritonitis by serotype 16 [1]. The diversity of infections caused by serotypes other than serotype 2 is indicative of the awareness of diagnosticians of *S. suis* infections [1].

Phenotypically, *S. suis* resembles the viridans streptococcal species *Streptococcus gordonii*, *Streptococcus sanguinis* and *Streptococcus parasanguinis*, and therefore may be misidentified in many human diagnostic laboratories [4]. Therefore, molecular techniques as well as antiserum serotyping are needed for confirmation of *S. suis* as well as its serotype. We report here a case of sepsis caused by *S. suis* serotype 31 in a patient in Thailand, using a polyphasic approach to identify this bacterium, as well as the results of genetic characterization of putative virulence gene profile, capsule gene (*cps*) locus, and genetic relatedness of *S. suis* serotype 31. The information from this study may increase awareness of the emergence of *S. suis* infections caused by uncommon strains, which will be important in the development of surveillance systems and epidemiological control and prevention strategies.

## Case presentation

A 55-year-old man was admitted to a hospital in Suphanburi Province, a central region in Thailand on December 8, 2012. He had an underlying condition with liver cirrhosis from a history of hepatitis B infection and alcohol misuse. Before admission, he had a history of raw pork product consumption (4 days prior to onset). Physical examination revealed a temperature of 38.6 °C, pulse rate of 102 beats/min, respiratory rate of 23 breaths/min, and blood pressure of 110/65 mmHg. No nuchal stiffness, ecchymosis, or hearing loss was found. Initial diagnosis in this case was sepsis based on systemic inflammatory response syndrome [5]. His white blood cell count was 13,600 cells/μL (70 % neutrophils) and platelet count was 129,000/mm^3^. A comprehensive metabolic panel revealed elevated creatinine level (1.95 mg/dL), serum albumin level of 3.4 mg/dL, blood urea nitrogen (BUN) level of 18 mg/dL, serum aspartate aminotransferase (AST) level of 660 IU/L, serum alanine aminotransferase (ALT) level of 130 IU/L, and creatine phosphokinase (CPK) level of 740 U/L. A bacterial isolate (no. 43640) was separated from the blood of the patient and identified as viridans group *Streptococcus* at the hospital laboratory. The isolate was sent to a reference laboratory for confirmation of its species. It was identified as *S. suis* by biochemical tests, multiplex polymerase chain reaction (PCR), and 16S rRNA gene sequencing (99 % identity) [6–8]. Based on these results, this case was finally diagnosed as sepsis. The patient was treated with ceftriaxone and discharged on December 14, 2012.

**Fig. 1:**
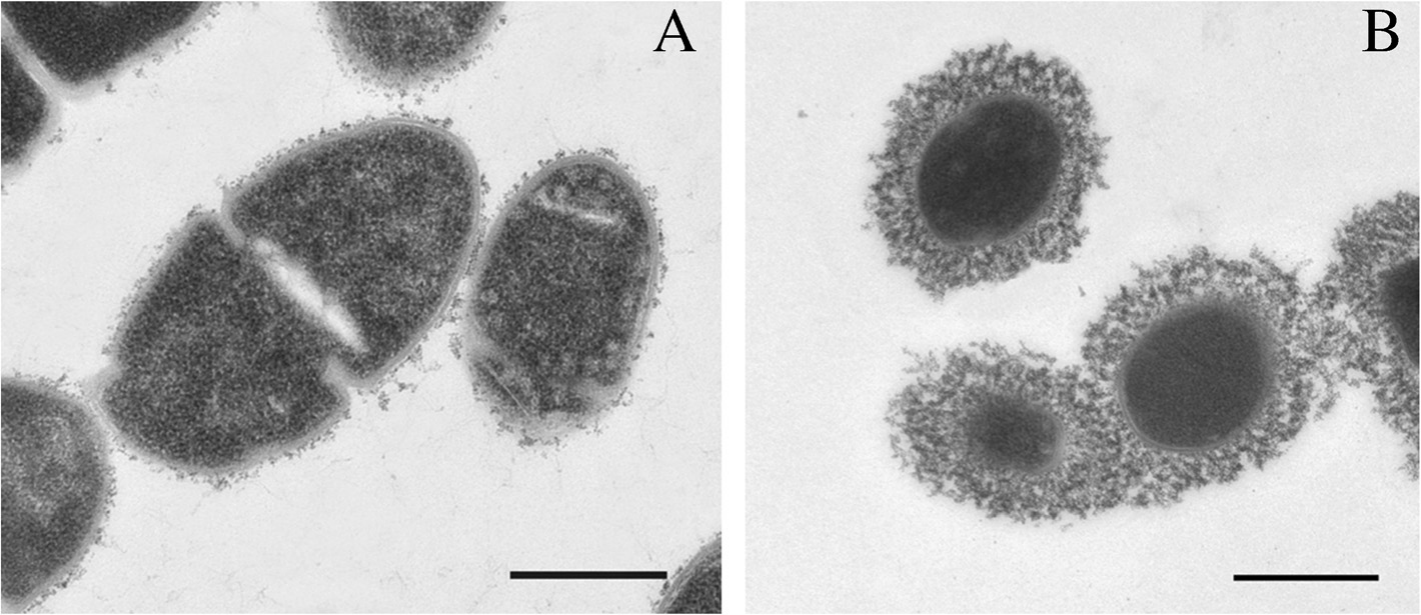
Transmission electron micrographs of unencapsulated *S. suis* isolate no. 43640 (**a**), compared with an encapsulated strain (**b**). Scale bar = 0.5 μm

Multiplex PCR demonstrated that this isolate belonged to serotype 31; however, antiserum serotyping revealed that this strain did not agglutinate with the antisera of any recognized *S. suis* serotypes [7, 9]. Further analysis of this strain by transmission electron microscopy confirmed almost complete absence of capsular material around the bacterial cells (Fig. 1) [10], showing the isolate to be unencapsulated. The isolate was susceptible to penicillin (MIC ≤0.12 μg/mL), ceftriaxone (MIC ≤1 μg/mL), erythromycin (MIC ≤0.25 μg/mL), levofloxacin (MIC ≤2.0 μg/mL), clindamycin (MIC ≤0.25 μg/mL), and vancomycin (MIC ≤1.0 μg/mL), and was resistant to tetracycline (MIC ≥8.0 μg/mL). Since breakpoints for *S. suis* are not defined in the 2014 Clinical and Laboratory Standards Institute guidelines, breakpoints for the viridans group streptococci were used instead.

**Fig. 2:**
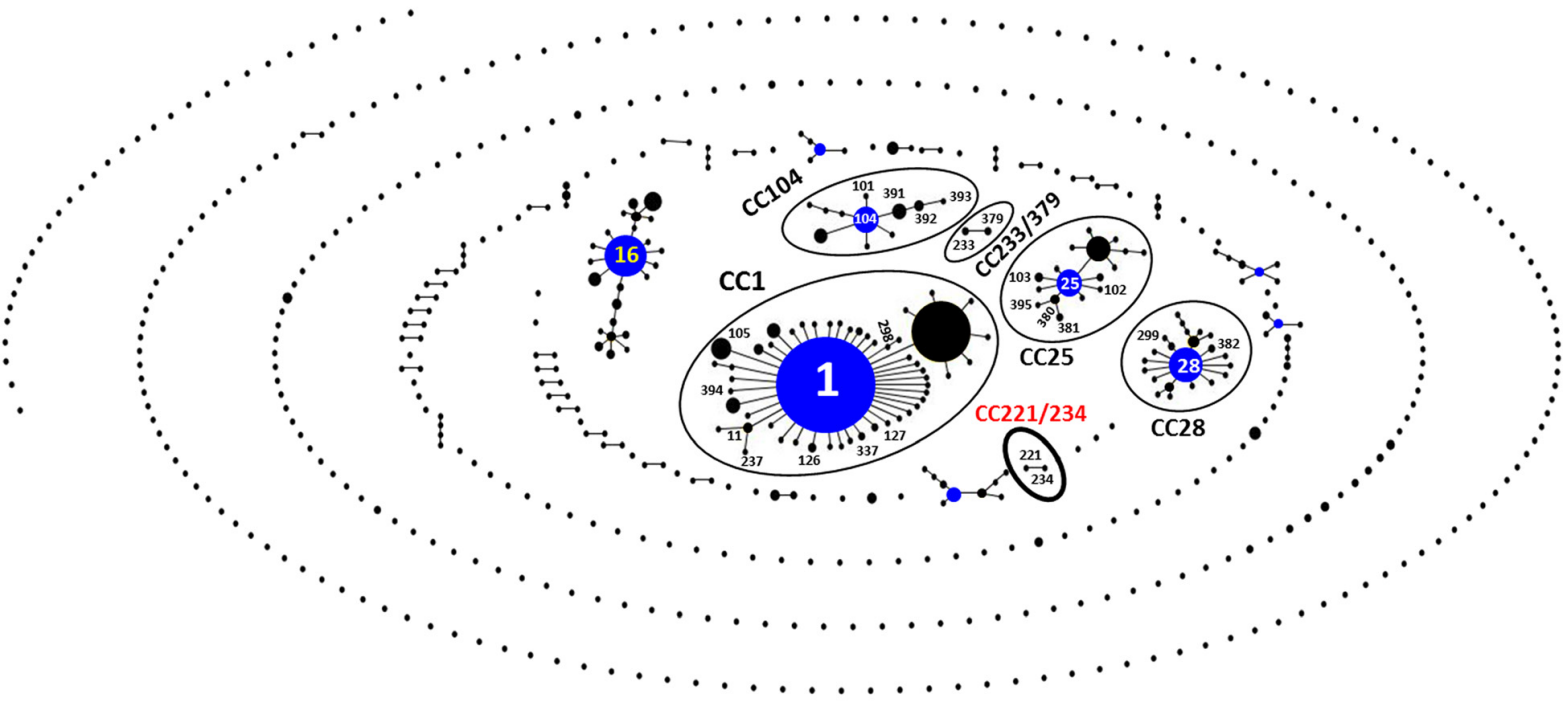
An eBURST analysis of the entire *S. suis* MLST database (accessed on May 15, 2015). Clonal complexes relevant to human infection in Thailand are circled and labeled. *S. suis* serotype 31 (ST221) in this study belonged to CC221/234 (bold circle). Clonal complexes and the predicted founders STs are indicated by blue dots. The size of the dots is relative to the number of isolates with the respective ST present in the database

Multilocus sequence typing (MLST) analysis of this isolate showed that it belonged to sequence type (ST) 221 of clonal complex (CC) 221/234 (Fig. 2) [11]. PCR and sequencing were used to study the presence of 29 genes previously associated with *S. suis* serotype 2 virulence. These genes include the muramidase-released protein gene (*mrp*), the extracellular factor gene (*epf*), the suilysin gene (*sly*), the arginine deaminase gene (*arcA*) [12], the factor H-binding surface protein gene (*fhb*) [13], the fibronectin-binding protein gene (*fbps*) [14], an infection-related factor gene (*trag*) [15], the serum opacity factor gene (*ofs*) [16], the S-ribosylhomocysteinase gene (*luxS*) [17], the hyaluronate lyase gene (*hyl*) [18], the glutamine synthetase gene (*glnA*) [19], an amylopullulanase gene (*apuA*) [20], an enolase gene (*eno*) [21], an IgA protease gene [22], the subtilisin-like protease gene (*sspA*) [23], the sortase A gene (*srtA*) [24], the sortase BCD gene (*srtBCD*), the sortase E gene (*srtE*), the sortase F gene (*srtF*), the sortase G gene (*srtG*) [25], the zinc uptake regulator gene (*zur*) [26], the transcriptional regulator gene (*rgg*) [27], orphan response regulator genes including *covR* and *revS* [28, 29], the two-component regulatory system gene (*ciaRH*) [30], the divalent-cation-related ABC transporter genes (*adcR* and *fur*) [31], an iron-transporter gene (*feoB*) [32], and the virulence-related gene (*virA*) [33]. Our analysis of this isolate revealed the presence of 13 genes found in virulent serotype 2 strains. The IgA protease gene, *arcA*, *luxS*, *glnA*, *apuA*, *eno*, *sspA*, *srtA*, *covR*, *zur*, *fur*, *adcR* and *feoB* were present in this isolate; however, the well-recognized virulence markers (*epf*/*mrp*/*sly*) were absent.

**Fig. 3:**
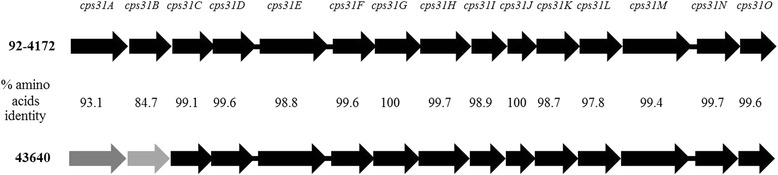
Genetic organization of the *cps* locus in unencapsulated *S. suis* serotype 31 isolate no. 43640 and reference *S. suis* serotype 31 strain 92-4172. Gray arrows indicate low similarity of amino acid sequences. Black arrows show high similarity of amino acid sequences

The entire *cps* locus of the unencapsulated serotype 31 strain 43640 (accession number KM576773) as well as *cpsA* and *cpsB* of five encapsulated *S. suis* serotype 31 strains (p523, p346, p369, p567 and p559; accession numbers KM884773–KM884777) isolated from slaughtered pigs were amplified and sequenced [see Additional file 1: Table S1 and Additional file 2: Table S2]. Our isolate showed an intact *cpsA-cpsO* (Fig. 3) as described for serotype 31 reference strain 92–4172 (accession number AB737835). No insertions, deletions, or frameshifts were found in the genes of the *cps* locus. In addition, the nucleotide sequence of *cpsA-cpsO* in this isolate showed a high identity (97 %) with the reference serotype 31. Comparison of the CpsA–CpsO protein sequences of both strains also revealed high identities (97.75–100 %), except for the CpsA and CpsB proteins, which had lower identities (93.11 and 84.71 %, respectively) with proteins of the reference strain (Fig. 3).

**Fig. 4:**
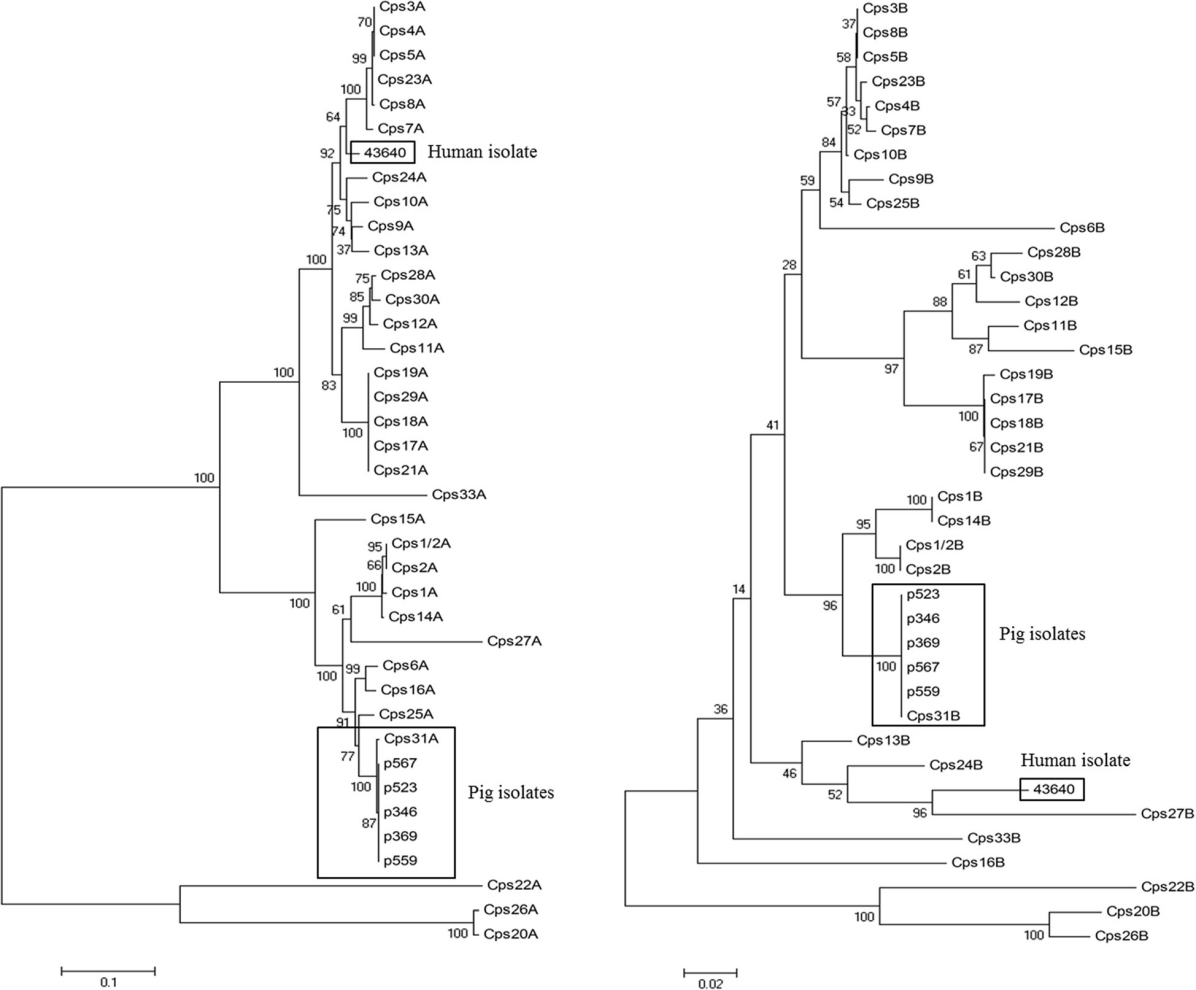
Unrooted tree based on the alignments of CpsA (left) and CpsB (right) amino acid sequences in 33 serotypes of *S. suis* and our isolates by a neighbor-joining method. Numbers at nodes represent bootstrap support expressed in percentages (1000 replications). Scale bar indicates sequence dissimilarity

Following the analysis of *cpsA* and *cpsB*, the CpsA protein of the unencapsulated strain 43640 was found to be composed of 479 amino acids with the highest identity to serotype 9-CpsA (96.66 %), followed by serotype 10-CpsA (96.45 %) and serotype 24-CpsA (95.62 %) (Fig. 4). The CpsB protein sequence comprised 229 amino acids that were identical to the reference serotype 31 as well as other serotypes. The sequence presented a greater identity to CpsB of the reference serotype 13 (91.27 %) and 24 (91.27 %) strains than to the reference serotype 31 (84.71 %) strain (Fig. 4). As shown in Fig. 4, the CpsA and CpsB sequences of five additional encapsulated *S. suis* serotype 31 strains isolated from pigs clustered with the reference serotype 31 strain with a high percentage identity (99–100 %), even though these sequences in unencapsulated strain no. 43640 were distant.

## Discussion

This is believed to be the first report of an unencapsulated *S. suis* serotype 31 infection in humans. In general, identification of *S. suis* as well as its serotypes can be achieved with a polyphasic approach using biochemical testing, antisera serotyping, and/or PCR assays [1]. As mentioned above, human laboratories are less able to identify this organism because of limited resources/technology, knowledge of *S. suis*, or phenotypic resemblance between *S. suis* and the viridans streptococcal species or others. Our study demonstrated that the hospital laboratory misidentified *S. suis* as viridians group *Streptococcus*. Even though Thailand has a reference laboratory to confirm unidentified bacteria from hospitals using a polyphasic approach, laboratory capacity should be increased, especially in hospital laboratories, to increase their knowledge of *S. suis* identification.

The strain isolated from this case belonged to ST221 in CC221/234. To the best of our knowledge, only two other unencapsulated strains have been recovered from human cases [3, 34], although unencapsulated *S. suis* has frequently been isolated from pigs [1, 35]. It is particularly interesting that CC221/234 did not contain any serotype 2 strains, but rather only serotype 24 strains (ST221 and ST234) (http://ssuis.mlst.net/sql/burstspadvanced.asp). This CC is not related to other CCs, especially the CCs that are associated with human and pig infections, such as CC1, CC16, CC20, CC25, CC27 and CC104 (Fig. 2) [1, 11, 36]. We have shown that CC221/234 *S. suis* serotype 24 can cause sepsis, similar to the isolate in this study [2]. This suggests that CC221/234 is a newly emerging, human infectious clone that does not include serotype 2 strains.

Our isolate revealed the presence of 13 putative virulence genes in virulent serotype 2 strains, as described above. The presence of one or more of these genes may have contributed to the virulence of this strain. Published evidence indicates that mutations in one or more of these putative virulence genes are sufficient to decrease the virulence of *S. suis*. For example, ApuA promotes adhesion to porcine epithelium and mucus *in vitro* [20]. The subtilisin-like protease (*sspA*) and the IgA protease are able to degrade natural proteins in host cells, for example gelatins, fibrinogens, and IgA antibody [22, 23]. Enolase plays an important role in adhesion to and invasion of brain microvascular endothelial cells [21]. Aranda et al. [32] demonstrated that full virulence of *S. suis* requires the FeoB transporter. Inactivation of *glnA* in *S. suis* resulted in reduced mortality and morbidity in murine infection models [19]. In addition, inactivation of *zur*, *covR* and *luxS* interferes with the expression of virulence factors, significantly contributing to attenuation of the virulence of *S. suis* [17, 26, 28].

The present isolate caused sepsis with signs and symptoms that did not differ from those of infection with *S. suis* serotype 2 strains. However, it is particularly interesting that patients with infections caused by bacteria other than serotype 2 also have predisposing conditions such as cirrhosis, asplenia and splenectomy: a likely explanation is that these infections are the result of immunocompromise [1–3, 34, 37]. The fact that the strain analyzed in this study was unencapsulated makes it even more atypical. A previous study reported that loss of the capsule may increase bacterial adhesion to host cells, increase biofilm formation that may allow bacteria to become persistent colonizers and to resist clearance by the host immune system and antibiotics, as well as induce the secretion of cytokines such as tumor necrosis factor-α, interleukin (IL-1β, IL-6, and IL-8) [10, 38–40]. Loss of the capsule may exacerbate the immunological response induced by the exposed cell wall components, leading to uncontrolled inflammatory reactions that may promote the development of a severe disease outcome. Moreover, alcohol abuse and liver cirrhosis may have contributed to the impaired neutrophil function in this patient [41, 42]. These observations may explain why the unencapsulated strain could survive in the blood circulation and cause disease.

Three hypotheses have been established to explain the unencapsulated phenotype. (1) Mutation in the promoter region of the *cps* locus may contribute to unencapsulation. Indeed, it has been shown that *Staphylococcus aureus* strains with mutations in the promoter region upstream of *cap5*(*8)A* fail to transcribe the capsule genes [43]*.* (2) Some amino acid substitutions that are not due to nonsense mutations and seem to result in an intact protein may also contribute to unencapsulation [44]. That study demonstrated that a single amino acid substitution each in Cps2E and Cps2F was the main cause of the capsule loss in *S. suis* serotype 2 or 1/2 isolates [44]. In the comparison between our isolate and reference serotype 31 strains, amino acid substitutions were found in the CpsA–CpsO protein sequences; only CpsG and CpsJ were found to have 100 % identity in their amino acid sequences. CpsE and CpsF of this unencapsulated strain revealed seven and one amino acid substitutions, respectively. Other protein sequences had substitutions in one position of CpsD, CpsH, CpsI, CpsN and CpsO; two positions of CpsC; three positions of CpsI; five positions of CpsK and CpsM; and nine positions of CpsL (data not shown). (3) *S. suis* capsules are believed to be synthesized via the Wzx/Wzy-dependent pathway, with a mature capsule translocated to the cell wall by the Wzd/Wze protein complex encoded by *cpsB* and *cpsC* [45]. In our unencapsulated strain, the *cpsA* and *cpsB* genes that encoded the CpsA and CpsB proteins had low similarity to those of the reference serotype 31 strain (Figs. 3 and 4). We hypothesize that dissimilar CpsB proteins may affect the compatibility of the protein–protein interactions for capsule processing in this unencapsulated strain. While CpsA is a capsule expression regulator, it appears to modulate the transcription of the capsule mRNA through specific binding to the capsule gene promoter [46, 47]. The dissimilarity of this protein in our isolate and in reference serotype 31 may also result in reduced capsule expression. Incompatibility of the protein–protein or protein–DNA interaction cannot be ruled out.

Similar to our study, Liu et al. [48] reported that the *cps* genes of *S. suis* strain YS54 were similar to reference serotype 29 strain 92-1191, except for *cpsH* and *cpsI*. The *cpsH* and *cpsI* of YS54 were similar to those of reference serotype 21 strain 14A, while the *cpsH* and *cpsI* of strain 14A shared no similarity with strain 91-1191 [48]. We speculated that horizontal gene transfer (HGT) may have been involved in *cpsA-cpsB* replacement/switching in our case; however, we do not know the source of the genes. HGT, via transformation, transduction or conjugation, can lead to the acquisition of entirely new sequences, as well as sequences that are homologous to existing DNA. The transfer of DNA via homologous recombination leads to the replacement of a region of the genome of a recipient cell by the corresponding region from the donor cell [49]. HGT events are frequently observed in *Streptococcus* such as *Streptococcus pyogenes*, *Streptococcus agalactiae* and *Streptococcus pneumoniae*; for example, genetic studies of *S. pneumoniae* serotype 6A and 6C capsule gene loci showed that substitution of *wciN6C* for *wciN6A* through homologous recombination resulted in a serotype switch from 6A to 6C [50].

## Conclusion

Although the isolation rate for this bacterial strain is still low, the emergence of *S. suis* infections caused by uncommon serotypes as well as unencapsulated strains should raise awareness in patients with predisposing conditions. Increased laboratory capacity to identify *S. suis* in hospitals is necessary in Thailand as well as in developing countries to contribute towards enhanced surveillance, epidemiological control, and prevention strategies for public health. Further investigations, such as complementation assay, should be considered to demonstrate clearly the mechanisms of capsular loss in this particular isolate [44].

## Consent

Written informed consent was obtained from the patient for publication of this Case report. A copy of the written consent is available for review by the Editor of this journal.

## Additional files

Additional file 1: Table S1. Primer sets used for PCR and sequencing of *cps* locus of the unencapsulated *S. suis* serotype 31 strain 43640. (DOCX 25 kb) Additional file 2: Table S2. Primer sets used for PCR and sequencing of *cpsA* and *cpsB* of the encapsulated *S. suis* serotype 31 isolated from pigs in this study. (DOCX 18 kb)
