# Supplementary material for: First human case report of sepsis due to infection with Streptococcus suis serotype 31 in Thailand
Source: BMC Infect Dis. 2015 Sep 30;15:392. doi: 10.1186/s12879-015-1136-0 (PMC4588491; doi:10.1186/s12879-015-1136-0)
Supplement: Additional file 1: Table S1. — Primer sets used for PCR and sequencing of cps locus of the unencapsulated S. suis serotype 31 strain 43640. (DOCX 25 kb) [file 12879_2015_1136_MOESM1_ESM.docx]

**Additional file 1: Table S1.** Primer sets used for PCR and sequencing of *cps* locus of the unencapsulated *S. suis* serotype 31 strain 43640.

| **Reaction no.** | **Primer** | **Sequence (5′-3′)** | **Primer**  **binding sites*** | **Region of *cps* locus** | **Product size (bp)** |
| --- | --- | --- | --- | --- | --- |
| 1 | 1-F | CTAGAGAATGCCTCATTTTATCCCC | 2963-2987 | *yaaA-cps31C* | 3,125 |
|  | 1-R | GCGAGACTAGCCGCAGTTCTAC | 6067-6088 |  |  |
| 2 | 2-F | ATGGCAACGTTAGAAATTGCACG | 5919-5941 | *cps31C* | 684 |
|  | 2-R | TCATTTTCTGCCGTAATTTCCGTA | 6579-6602 |  |  |
| 3 | 3-F | GCGCAGAAATGTGATGCGATGG | 6414-6435 | *cps31C*-*cps31D* | 480 |
|  | 3-R | CTATAATACAATTCAGCACC | 6874-6893 |  |  |
| 4 | 4-F | ATGATTGATATTCATTCGCATATC | 6643-6666 | *cps31D* | 732 |
|  | 4-R | TTAAATCGCCTGGTTTTTTAATAGC | 7350-7374 |  |  |
| 5 | 5-F | CTGAAGCCCGCTTTAATTGGCG | 7144-7165 | *cps31D*-*cps31E* | 1,387 |
|  | 5-R | AACATGCTTATGCGCTGCAGC | 8511-8531 |  |  |
| 6 | 6-F | ATGGATTTAGGAACTGTTACCGA | 7404-7426 | *cps31E* | 1,827 |
|  | 6-R | TTATTCTTGTTTTGCAAATTC | 9210-9230 |  |  |
| 7 | 7-F | TTCTTTAAATGGTAAGGAGAG | 8063-8083 | *cps31E*-*cps31F* | 1,637 |
|  | 7-R | GAATCATTTAAGGGGTTGAC | 9680-9699 |  |  |
| 8 | 8-F | ATGGGACTAACATTAATGTATATTAC | 9473-9498 | *cps31F*-*cps31G* | 1,424 |
|  | 8-R | TTAAACCGTTTTATTCGTATTAATA | 10872-10896 |  |  |
| 9 | 9-F | CTGACGAATGGTGTAGTGGCAG | 9953-9974 | *cps31F-cps31H* | 1,370 |
|  | 9-R | GGCAACCCCAATTAACCC | 11305-11322 |  |  |
| 10 | 10-F | ATGTCACAGTTGCATTTATCAATAG | 10906-10930 | *cps31H* | 1,002 |
|  | 10-R | TTAATCTTCAAAAATACGAACAAATTC | 11881-11907 |  |  |
| 11 | 11-F | TGAAGATATTCCAGAAGGTTGG | 11490-11511 | *cps31H-cps31I* | 747 |
|  | 11-R | GAATGTTCATAGACAATAACAG | 12215-12236 |  |  |
| 12 | 12-F | TTGGATAGTATGAAAAAAGTTAATAC | 11923-11948 | *cps31I* | 846 |
|  | 12-R | TTAATTTAAAAACTCGAAATCAAAATC | 12742-12768 |  |  |
| 13 | 13-F | TTTCTTCCAAAGATTCTATAAAGC | 12435-12458 | *cps31I-cps31J* | 685 |
|  | 13-R | ATCGAAACATTTGGTAAAATAG | 13098-13119 |  |  |
| 14 | 14-F | ATGTATTTATTGATGAGATTGTTC | 12797-12820 | *cps31J* | 531 |
|  | 14-R | CTATTTTTTAGACTCAAATAGACTCC | 13302-13327 |  |  |
| 15 | 15-F | GCTGCTGGTGCTCTTGTTGCAAGC | 13139-13162 | *cps31J-cps31L* | 1,963 |
|  | 15-R | CCAGAACTAAAACAGAAG | 15084-15101 |  |  |
| 16 | 16-F | TTGAAAATGAAAAAAGTTTTATTAA | 13354-13378 | *cps31K-cps31L* | 2,348 |
|  | 16-R | TCATTTTGTCCATCCATGAATCTG | 15668-15691 |  |  |
| 17 | 17-F | ATTTGGAATAGGAAATGAGCT | 13947-13967 | *cps31K-cps31M* | 2,232 |
|  | 17-R | GTCGCAAGATATCTTTTCTGATC | 16156-16178 |  |  |
| 18 | 18-F | TCAAATACGTCTAGTACTGGC | 15271-15291 | *cps31L-cps31M* | 1,400 |
|  | 18-R | GAGACCGATATAATGCAATC | 16651-16670 |  |  |
| 19 | 19-F | ATGGAAAAACAGAGTAGAACAAG | 15694-15716 | *cps31M* | 1,560 |
|  | 19-R | CTAGCTCCTACTAGTTTTCAAGTA | 17230-17253 |  |  |
| 20 | 20-F | TTTTAATTTCAAGGAAAGCG | 16298-16317 | *cps31M-cps31N* | 1,734 |
|  | 20-R | CAAGATAATTCCGACAGCAGG | 18011-18031 |  |  |
| 21 | 21-F | TGATAGGTACAAAGCATTGGCCG | 16827-16849 | *cps31M-cps31O* | 1,744 |
|  | 21-R | TTCTCTAATCACATCTTGACG | 18550-18570 |  |  |
| 22 | 22-F | GGAATCTGCAGAAAACAGACTACG | 18306-18329 | *cps31N-cps31O* | 787 |
|  | 22-R | TCAAATACTGTTATTTTTCACGAAAG | 19067-19092 |  |  |
| 23 | 23-F | GGGTGAATATATTGGTGCAGC | 18855-18875 | *cps31O-glf* | 989 |
|  | 23-R | GCGCCATATTCATGGACTTG | 19823-19842 |  |  |
| - | Seq1** | CCGACAGAAGTGATGACCAGT | - | *cps31A-cps31B* | - |
| - | Seq2** | ATGAAGAAGAGAAGCAGATGG | - | *cps31A-cps31B* | - |
| - | Seq3** | CCATTAGCCAACGAATACCGCTC | - | *cps31A-cps31B* | - |
| - | Seq4** | GTTGATTGATTTGGTTGGTGG | - | *cps31A-cps31B* | - |

* The primer binding sites were based-on the nucleotide position in *cps* locus of reference *S. suis* serotype 31 strain 92-4172.

** These primers were specifically designed to sequencing of *cpsA-cpsB* in *cps* locus of *S. suis* serotype 31 strain 43640 from PCR product of reaction no. 1.
