# Supplementary material for: First human case report of sepsis due to infection with Streptococcus suis serotype 31 in Thailand
Source: BMC Infect Dis. 2015 Sep 30;15:392. doi: 10.1186/s12879-015-1136-0 (PMC4588491; doi:10.1186/s12879-015-1136-0)
Supplement: Additional file 2: Table S2. — Primer sets used for PCR and sequencing of cpsA and cpsB of the encapsulated S. suis serotype 31 isolated from pigs in this study. (DOCX 18 kb) [file 12879_2015_1136_MOESM2_ESM.docx]

**Additional file 2: Table S2.** Primer sets used for PCR and sequencing of *cpsA* and *cpsB* of the encapsulated *S. suis* serotype 31 isolated from pigs in this study.

| **Primers** | **Sequence (5′-3′)** | **Primer binding sites** | **Region of *cps* locus** | **Product size (bp)** | **Purpose** |
| --- | --- | --- | --- | --- | --- |
| SS31-F | GATAACGTTTTCTGATGTTGAGCG | 3668-3691 | Upstream of *cps31A*-*cps31C* | 2,420 | PCR amplification and sequencing |
| SS31-R | GCGAGACTAGCCGCAGTTCTAC | 6067-6088 |  |  |  |
| SS31-seq1 | TTATTTTTCAACAAGTACAGACTG | 5179-5202 | *cps31A* | - | Sequencing |
| SS31-seq2 | CTATTTCAATTTCTTCGAATCTGG | 5886-5909 | *cps31B* | - | Sequencing |
